# The Practical Medicine Series of Year Books

**Published:** 1903-04

**Authors:** 


### Books and Magazines.

**All books intended for review in the Texas Medical Journal must be sent to Associate Editor Dr. W. B. Russ, San Antonio, Texas.**

---

THE PRACTICAL MEDICINE SERIES OF YEAR BOOKS, Comprising the volumes of the Year's Progress in Medicine and Surgery. Issued monthly under the general editorial charge of Gustavus P. Herd, M. D., Professor of Laryngology and Rhinology, Chicago Post-Graduate Medical School. The Year Book Publishing Co., 40 Dearborn Street, Chicago. $1.25 each, or $7.50 per year.

Primarily, this series is intended for the general practitioner and presents a comprehensive review of the year in medicine.

The reviewer has received the volumes devoted to General Surgery, edited by John B. Murphy, M. D., Chicago; Obstetrics, edited by R. Peterson, M. D., of the University of Michigan, and H. F. Lewis, M. D., of the Rush Medical College; Materia Medica and Therapeutics, Preventive Medicine, Climatology and Forensic Medicine, edited by George F. Butler, M. D., H. B. Favill, M. D., N. Bridge, M. D., and H. N. Moyer, M. D.; Pediatrics and

Orthopedic Surgery, edited by W. S. Christopher, M. D. John Ridlon, M. D., and Sam J. Walker, M. D.; Physiology, Pathology, Bacteriology and Anatomy, edited by W. O. Evans, M. D., and Adolph Gehrman, M. D.; General Medicine, edited by Frank Billings, M. D., and J. H. Salisbury, M. D. T. J. B.

